# Exogenous Bio-Based 2,3-Butanediols Enhanced Abiotic Stress Tolerance of Tomato and Turfgrass under Drought or Chilling Stress

**DOI:** 10.4014/jmb.2201.01025

**Published:** 2022-04-18

**Authors:** Ae Ran Park, Jongmun Kim, Bora Kim, Areum Ha, Ji-Yeon Son, Chan Woo Song, Hyohak Song, Jin-Cheol Kim

**Affiliations:** 1 Department of Agricultural Chemistry, Institute of Environmentally Friendly Agriculture, College of Agriculture and Life Sciences, Chonnam National University, Gwangju 61186, Republic of Korea; 2 Plant Healthcare Research Institute, JAN153 Biotech Incorporated, Gwangju 61186, Republic of Korea; 3 Research and Development Center, GS Caltex Corporation, Daejeon 34122, Republic of Korea

**Keywords:** 2,3-Butanediol, abiotic stress, formulation, signaling pathway-related genes

## Abstract

Among abiotic stresses in plants, drought and chilling stresses reduce the supply of moisture to plant tissues, inhibit photosynthesis, and severely reduce plant growth and yield. Thus, the application of water stress-tolerant agents can be a useful strategy to maintain plant growth under abiotic stresses. This study assessed the effect of exogenous bio-based 2,3-butanediol (BDO) application on drought and chilling response in tomato and turfgrass, and expression levels of several plant signaling pathway-related gene transcripts. Bio-based 2,3-BDOs were formulated to levo-2,3-BDO 0.9% soluble concentrate (levo 0.9% SL) and meso-2,3-BDO 9% SL (meso 9% SL). Under drought and chilling stress conditions, the application of levo 0.9% SL in creeping bentgrass and meso 9% SL in tomato plants significantly reduced the deleterious effects of abiotic stresses. Interestingly, pretreatment with levo-2,3-BDO in creeping bentgrass and meso-2,3-BDO in tomato plants enhanced JA and SA signaling pathway-related gene transcript expression levels in different ways. In addition, all tomato plants treated with acibenzolar-S-methyl (as a positive control) withered completely under chilling stress, whereas 2,3-BDO-treated tomato plants exhibited excellent cold tolerance. According to our findings, bio-based 2,3-BDO isomers as sustainable water stress-tolerant agents, levo- and meso-2,3-BDOs, could enhance tolerance to drought and/or chilling stresses in various plants through somewhat different molecular activities without any side effects.

## Introduction

Drought and chilling damage are important abiotic stresses that affect plant growth and productivity by reducing the supply of moisture and significantly inhibiting photosynthesis [1-3]. Economic losses from drought and chilling injuries can result directly from visible damage to the plant body and indirectly from reduced quality. To mitigate damages caused by abiotic stress, plants need to adapt, acclimatize, or modify the environmental stresses by stress escape, avoidance, or tolerance [4]. Biostimulants are substances or microorganisms (*e.g.*, beneficial bacteria) that, when used to treat seeds, plants, or the rhizosphere, support biological processes to enhance nutrient uptake, nutrient efficiency, tolerance to abiotic stress, or crop quality and yield. Especially, beneficial bacteria can provide sustainable and eco-friendly solutions to agricultural challenges [5, 6]. Although beneficial bacteria, such as plant growth-promoting rhizobacteria (PGPR), are applied primarily for managing plant pathogens, they are also increasingly used in conventional and organic farming practices to bolster plant growth and tolerance [7].

Bacteria with plant growth-promoting activity belong to several genera, including *Azotobacter*, *Bacillus*, *Klebsiella*, *Pseudomonas*, and *Rhizobium* [8-11]. The functional mechanisms of PGPR are varied and include the ability to change hormone content, produce volatile compounds, promote nutrient availability, or enhance abiotic stress tolerance [12]. Interactions between plants and rhizosphere microbes are known to increase tolerance to drought directly or indirectly [13]. Among PGPR, several bacteria, including *Bacillus* and *Klebsiella*, produce volatile organic compounds, such as 2,3-butanediol (2,3-BDO), which are known to induce plant systemic resistance against phytopathogens and can also enhance tolerance to abiotic stresses in plants [14].

In the past, 2,3-BDO was chemically manufactured. However, rising petroleum prices and an ever-increasing interest in renewable biomass have inspired state-of-the-art strategies for efficient microbial 2,3-BDO production through strain improvement, substrate alternation, and process development [15-17]. Also known as 2,3-butylene glycol, 2,3-BDO exists as three stereoisomers: (*2R*,*3R*)-2,3-BDO and (*2S*,*3S*)-2,3-BDO, known as levo-and dextro-2,3-butanediol, respectively, and (*2R*,*3S*)-2,3-BDO, known as meso-2,3-butanediol. The 2,3-BDO product can be altered through highly complicated strain-specific enzyme reactions. Strains of *Klebsiella* and *Enterobacter* are known to synthesize (*2S*,*3S*)-2,3-BDO and (*2R*,*3S*)-2,3-BDO, while members of *Bacillus* mainly synthesize (*2R*,*3R*)-2,3-BDO and (*2R*,*3S*)-2,3-BDO. GS Caltex, a commercial producer of bio-based 2,3-BDO, has reported that *Bacillus licheniformis* strain 4071 (isolated from the onion rhizosphere) produces (*2R*,*3S*)-BDO and (*2R*,*3R*)-BDO isomers [18, 19].

As a potential bulk chemical, 2,3-BDO has extensive industrial application in chemistry, cosmetics, agriculture, and pharmaceuticals [20-24]. However, although many studies have elucidated the role of 2,3-BDO in the industry, few studies have evaluated the various interactions of different stereoisomers of microbial 2,3-BDO in plant signaling pathways and their consequent effects on drought and chilling stress. Moreover, there have been few studies of formulating eco-friendly 2,3-BDOs produced by microorganisms and inducing abiotic stress tolerance in plants using 2,3-BDO formulations.

In the present study we aimed to evaluate the effects of two microbial 2,3-BDO formulations (levo 0.9% SL and meso 9% SL) on abiotic stresses and inform the effective use of 2,3-BDOs in agriculture. We developed two formulations of bio-based 2,3-BDO produced by *B. licheniformis* strain 4071, evaluated their effects through in vivo experiments, and analyzed gene expression in plants treated with 2,3-BDO formulations to elucidate the relationship with the plant hormone signaling pathway.

## Materials and Methods

### Sample Preparation and Analytical Procedure of Bio-Based 2,3-BDO

GS Caltex Corp. provided bio-based levo-2,3-BDO and meso-2,3-BDO samples with 99% purified (*2R*,*3R*)-2,3-BDO or (*2R*,*3S*)-2,3-BDO. Briefly, the samples were prepared via fermentation by *B. licheniformis* 4071, with separation and purification according to previous reports [18, 25]. Three stereoisomers of 2,3-BDO, (*2R*,*3R*)-2,3-BDO, (*2R*,*3S*)-2,3-BDO, and (*2S*,*3S*)-2,3-BDO, were determined by gas chromatography with flame ionization detector (GC-FID; HP 6890 series, Hewlett Packard, USA) equipped with an HP-chiral 20ß column (30 m, 0.32 mm internal diameter, 0.25 μm film thickness; Agilent Technologies, Germany). The oven temperature was initially set at 40°C for 5 min and increased to 160°C at a gradient of 15°C/min, at which point it was maintained for 2 min. The temperature of the injector and detector was set at 230°C. Argon was used as the carrier gas and run through the column at 2 ml/min. The sample injection volume was 0.2 μl.

### Formulation of Bio-Based 2,3-BDO

Bio-based (*2R*,*3R*)-2,3-BDO (0.9 g) was mixed with 1.0 g of ethoxylated C12-14 secondary alcohol (Softanol 90, Nippon Shokubai Co., Ltd, Japan) as an emulsifier, 30.0 g of propylene glycol monomethyl ether as a solvent, and 68.1 g of water to prepare a levo 0.9% soluble concentrate (SL) formulation. Bio-based (*2R*,*3S*)-2,3-BDO (9 g) was mixed with 5.0 g Softanol 90 (Nippon Shokubai Co. Ltd.) as an emulsifier, 30.0 g of propylene glycol monomethyl ether as a solvent, and 56.0 g of water to prepare a meso 9% SL formulation.

### Effect of 2,3-BDO Formulations on Drought Stress

Pot experiments were conducted to evaluate the effects of the levo 0.9% SL and meso 9% SL formulations against drought stress in tomato and turfgrass. Seokwang tomato seeds were sown in horticultural nursery soil at 25°C for 4 weeks. Tomato seedlings at the five-leaf stage were transplanted in 9.5-cm diameter pots containing a sterilized nursery soil-sand mix (1:1, v/v) and treated with 1,000-fold diluted solution (5 ml/pot) of levo 0.9% SL or meso 9%SL by foliar spray. Untreated and positive control plants were treated with 5 ml of distilled water and acibenzolar-S-methyl (ASM, 210 μg/ml; Syngenta Korea, Republic of Korea) by foliar spray, respectively. Creeping bentgrass (cultivar Penn A4) and Kentucky bluegrass seeds (0.25 g, respectively) were placed evenly within a pot (6 cm diameter, 8.5 cm height, 240 cm3 volume) filled with 100 g of dry sandy loam soil. Pots were incubated at 25°C in a culture room for 3 weeks. Following the 3-week growing period, a 1,000-fold diluted solution (10 ml) of levo 0.9%SL or meso 9% SL was added by drenching the soil. Untreated and positive control plants were treated with 10 ml of distilled water and ASM (210 μg/ml, Syngenta Korea) by soil-drenching.

To analyze the effect of drought tolerance according to treatment time, we treated one group of seedlings 7 days before drought stress and another group 7 days and 1 day before drought stress. Drought stress was conducted by ceasing irrigation in the tomato plants, creeping bentgrass, and Kentucky bluegrass. After 3 days, the survival rate and shoot weight of each plant were measured. Each treatment consisted of four tomato plants or turfgrass pots. The experiment was repeated twice. Drought tolerance was calculated as (survival rate of untreated control –survival rate of treatment)/survival rate of untreated control.

### Effect of 2,3-BDO Formulations on Chilling Stress

Pot experiments were conducted to evaluate the effects of levo 0.9% SL and meso 9% SL on chilling stress in tomato and turfgrass. We prepared the plants and treated them with 1,000-fold diluted solution of levo 0.9% SL or meso 9% SL as described in the drought stress experiment.

To analyze the effect of cold tolerance according to treatment time, we treated one group of seedlings 7 days before cold stress and another group 7 days and 1 day before chilling stress. Plants were exposed to chilling stress in a 4°C incubator 1 day after the second treatment. After 13 days of incubation, the survival rate was estimated and each plant was moved to a 25°C incubator. After 7 days, the survival rate was estimated again. Each treatment consisted of four tomato plants or turfgrass pots. The experiment was repeated twice.

### Analysis of Gene Expression Levels by qRT-PCR

We assessed the effect of levo 0.9% SL and meso 9% SL on the expression of four defense-related genes in potted tomato plants and creeping bentgrass using quantitative reverse-transcription (qRT)-PCR. In tomato plants, we estimated the levels of tomato phenylalanine ammonia-lyase (*LePAL*), allene oxide synthase 2 (*LeAOS2*), 1-aminocyclopropane-1-carboxylate synthase 4 (*LeACS4*), and peroxidase (*LePOX*) as proxies for salicylic acid (SA), jasmonic acid (JA), and ethylene (ET) signaling pathway-related genes and reactive oxygen species (ROS) scavenger gene, respectively [26-29]. In creeping bentgrass, we assessed the levels of creeping bentgrass nonexpresser of pathogenesis-related genes 1 (*AsNPR1*), lipoxygenase (*AsLOX*), ethylene response factor (*AsERF*), and peroxidase (*AsPOX*) as proxies for SA, JA, and ET signaling pathway-related genes and ROS scavenger gene, respectively [30-32].

The leaves of tomato and creeping bentgrass were harvested 1 and 2 days after treatment with 1,000-fold diluted levo 0.9% SL or meso 9% SL and their total RNAs were extracted using an RNeasy mini kit (Qiagen, USA) according to the manufacturer’s instructions. cDNA was prepared from 5 μg of total RNA with oligo (dT) primers and SuperScript IV reverse transcriptase (Invitrogen Inc., USA) according to the manufacturer’s protocols. The relative mRNA expression was determined in a real-time PCR detection system (Bio-Rad CFX 96; Bio-Rad Laboratories, USA). cDNA was analyzed using iQ SYBR Green Supermix (Bio-Rad Laboratories) in a 20 μl volume. The PCR primers of genes used in this study (Table 1) were synthesized by Genotech (Daejeon, Korea). Data were analyzed using BioRad CFX Manager version 2.1. Relative fold changes in mRNA between treatments were determined based on the ΔΔCT method with ubiquitin (*UBI*) as a housekeeping gene [24, 26]. Samples were analyzed in three biological replicates with three technical replicates and then averaged.

### Statistical Analysis

The parameters measured in this study were designed to evaluate abiotic stress-tolerant activity. Analyses were conducted separately for the pot experiments under drought and chilling stress conditions. All data were analyzed for the homogeneity of variance using SPSS statistical analysis software (version 21.0 for Windows; SPSS, USA). Data are expressed as the mean ± SD of replicates and evaluated by one-way analysis of variance. Significant differences among the treatments were determined according to Duncan’s multiple range test (*p* < 0.05).

## Results

### Analysis of Bio-Based 2,3-BDO

**Fig. 1:**
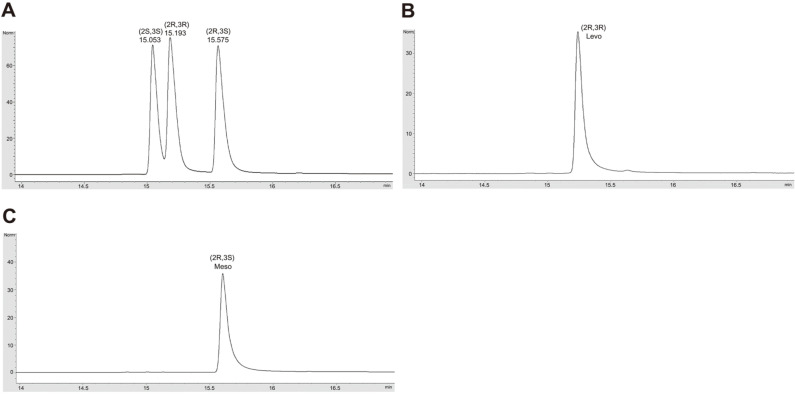
Gas chromatography of two bio-based 2,3-BDOs produced by *B. licheniformis* 4071. (**A**) A mixture of three stereoisomers: (*2R*,*3R*)-2,3-BDO, (*2R*,*3S*)-2,3-BDO, and (*2S*,*3S*)-2,3-BDO; (**B**) bio-based levo-2,3-BDO; and (**C**) biobased meso-2,3-BDO.

The result of gas chromatography of bio-based levo-2,3-BDO and meso-2,3-BDO provided by GS Caltex Corp. showed that they contained (*2R*,*3R*)-2,3-BDO and (*2R*,*3S*)-2,3-BDO with more than 99% selectivity, respectively (Fig. 1).

### Effect of 2,3-BDO Formulations on Drought Stress

**Fig. 2:**
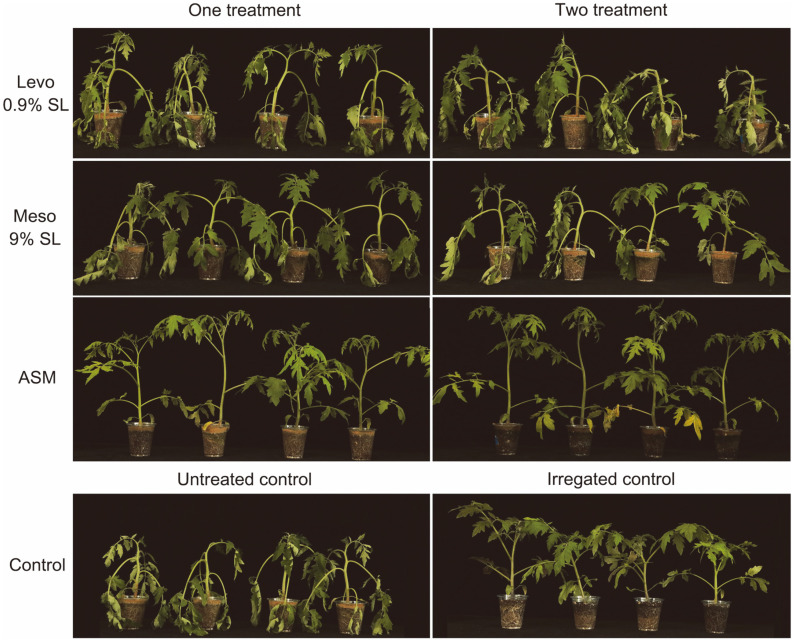
Effect of 2,3-BDO formulations on drought stress in tomato plants. Seedling groups were treated either 7 days before drought stress or 7 days and 1 day before drought stress.

Plant phenotypic assays were conducted 3 days after drought stress by ceasing irrigation in tomato plants and turfgrass. The leaves of the untreated control plants were largely wilted while those of ASM-treated plants remained turgid (Fig. 2). The drought tolerance value of the tomato plants treated with meso 9% SL was 50.0%with one treatment and 60.7% with two treatments compared to untreated control plants (Table 2). Meso 9% SL displayed much stronger drought tolerance than levo 0.9% SL in tomato plants. In addtion, the dry weights of levo and meso formulation-treated tomato plants were significantly higher than those of ASM-treated and untreated control tomato plants.

**Fig. 3:**
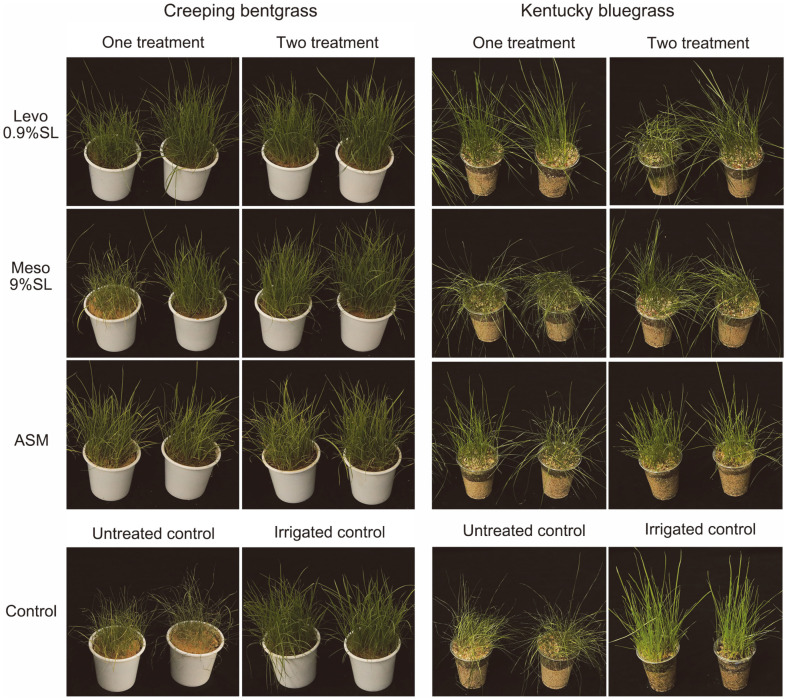
Effect of 2,3-BDO formulations on drought stress in creeping bentgrass and Kentucky bluegrass. Seedling groups were treated either 7 days before drought stress or 7 days and 1 day before drought.

Creeping bentgrass treated with levo 0.9% SL or meso 9% SL showed higher drought tolerance activity than ASM-treated plants (Table 3, Fig. 3). The drought tolerance activity values of levo 0.9% SL, meso 9% SL, and ASM were 86.7, 46.7, and 56.7% with one treatment, respectively, and 93.3, 96.7, and 50.0% with two treatments, respectively. The fresh and dry weights of 2,3-BDO formulations-treated and ASM-treated creeping bentgrass were significantly higher than the untreated control.

In Kentucky bluegrass, levo 0.9% SL or meso 9% SL exhibited higher drought tolerance activity than the untreated control. The drought tolerance value with one treatment of levo 0.9% SL was higher than those for ASM and meso 9% SL treatments. Among them, one treatment of levo 0.9% SL showed the highest drought tolerance value (89.4% compared to 48.4% after two treatments). The fresh weight with one treatment of levo 0.9% SL was also significantly higher than that of the untreated control.

### Effect of 2,3-BDO Formulations on Chilling Stress

**Fig. 4:**
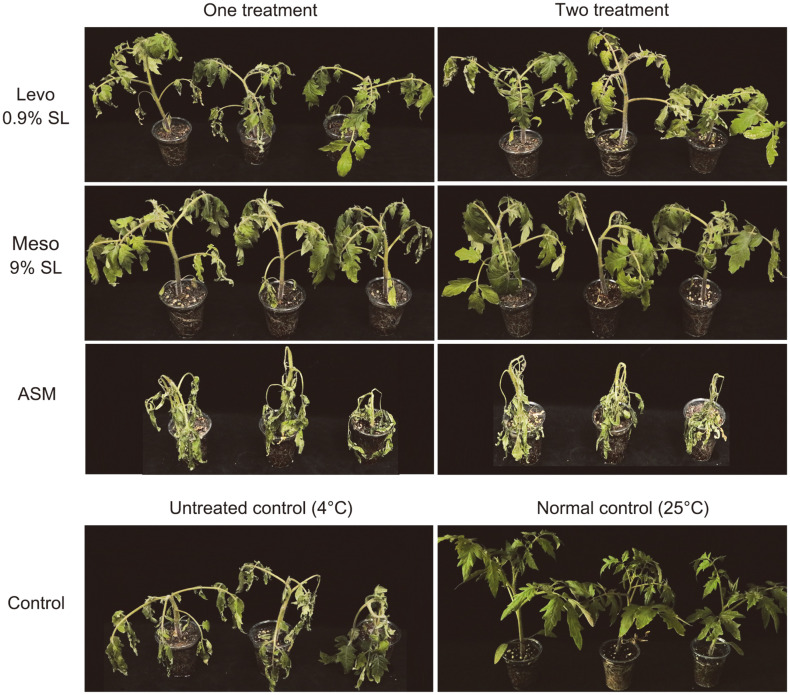
Effect of 2,3-BDO formulations on chilling stress in tomato plants. Seedling groups were treated either 7 days before chilling stress or 7 days and 1 day before chilling stress.

Significant wilting and curling were observed in untreated tomato plants after 13 days of chilling stress (Fig. 4). In tomato plants, two treatments of both levo 0.9% SL and meso 9% SL showed superlative tolerance effects compared to one treatment (Table 4). The cold tolerance values of levo 0.9% SL and meso 9% SL were 43.3 and 38.0% with one treatment, respectively, and 48.6 and 52.1% with two treatments, respectively. The tomato plant group treated with ASM suffered more severe cold damage than the untreated group.

**Fig. 5:**
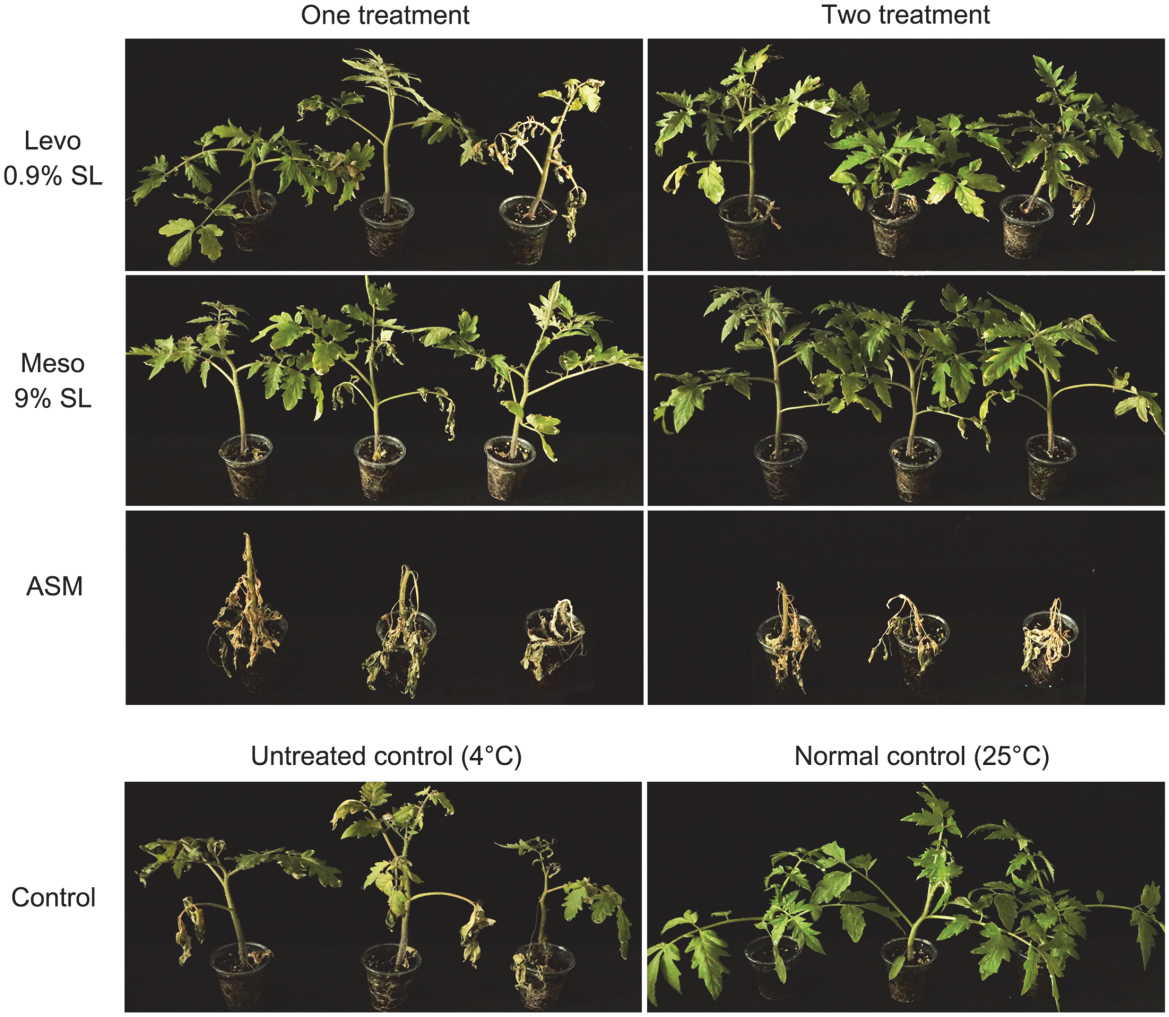
Effect of tomato plants by applying 2,3-BDO formulations 7 days after recovery from chilling stress.

After recovery at 25°C for 7 days, in both treatments of levo 0.9% SL and meso 9% SL, wilting caused by chilling stress was alleviated. Meso 9% SL showed recovery values of 76.3% with one treatment and 83.8% with two treatments. Levo 0.9% SL also improved recovery from cold stress, with tolerance values of 50.0% with one treatment and 76.3% with two treatments. After recovery, the phenotype of tomato plants treated with levo 0.9%SL and meso 9% SL and the untreated control plants recovered to some extent. However, all of the tomato plants treated with ASM were completely withered under chilling stress and did not recover at 25°C (Fig. 5). After recovery in normal conditions, the dry shoot weights of tomato plants also showed a similar trend to those of their cold tolerance, so that levo 0.9% SL- or meso 9% SL-treated tomato plants had higher dry shoot weight than the ASM-treated and the untreated groups.

**Fig. 6:**
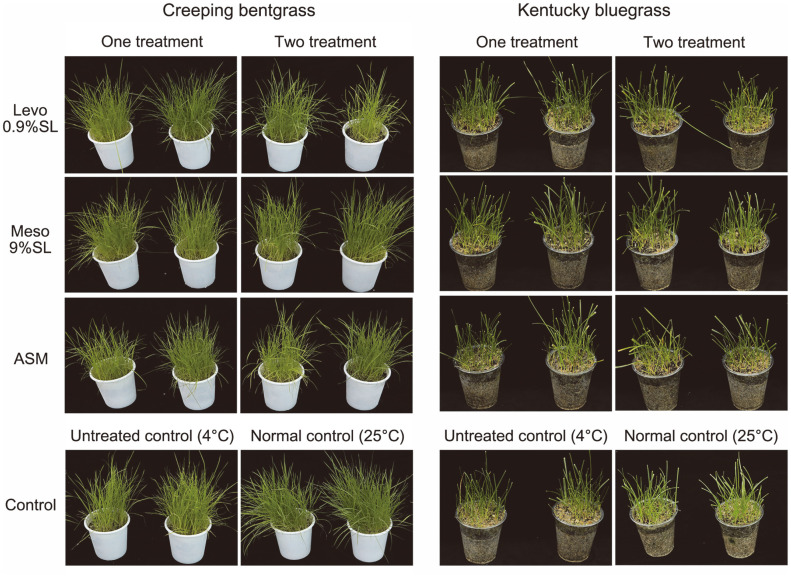
Effect of 2,3-BDO formulations on chilling stress in turfgrass. Seedling groups were treated either 7 days before chilling stress or 7 days and 1 day before chilling stress.

However, chilling stress did not cause any damage to creeping bentgrass and Kentucky bluegrass, with no significant difference in phenotype between the treatment groups (Table 5, Fig. 6).

### Analysis of Gene Expression Levels by qRT-PCR

**Fig. 7:**
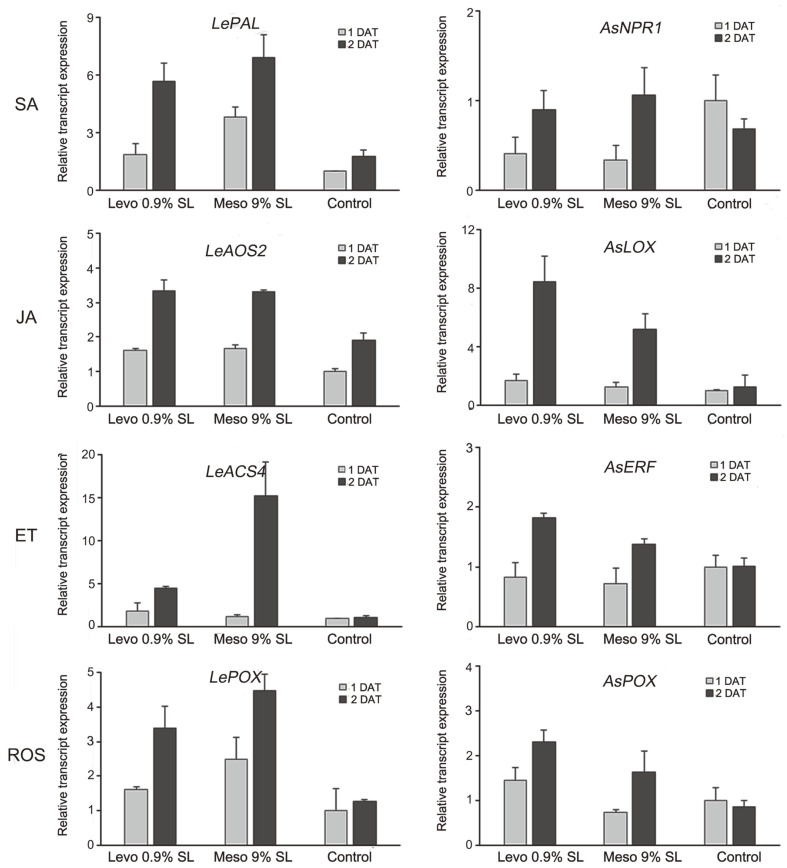
Quantitative reverse-transcription PCR of transcripts differentially expressed in tomato plants and creeping bentgrass after 2,3-BDO formulations treatments. The housekeeping gene ubiquitin (*UBI*) was used as the reference gene, and the data were calibrated relative to the transcript levels in the untreated control at 1 and 2 days after treatment. The data are presented as mean ± SD of three replicates.

The relative expression levels of SA pathway-related gene *LePAL*, JA pathway-related gene *LeAOS2*, ET pathway-related gene *LeACS4*, and ROS scavenger gene *LePOX* were higher in 2,3-BDO-treated tomato plants compared to those of the untreated controls (Fig. 7). The relative expression levels of *LePAL*, *LeACS4*, and *LePOX* were higher in meso 9% SL-treated tomato plants than in levo 0.9% SL-treated plants. At 1 day after treatment (DAT) and 2 DAT, a 3.79-fold and 3.92-fold increase in the expression of *LePAL* genes, 1.16-fold and 14.22-fold increase in the expression of *LeACS4* genes, and 2.49-fold and 3.52-fold increase in the expression of *LePOX* genes were observed in meso 9% SL-treated groups compared to those in the untreated control.

At 1 DAT, the relative expression levels of lipoxygenase gene *AsLOX*, ET pathway-related gene *AsERF*, and ROS scavenger gene *AsPOX* in creeping bentgrass treated with levo 0.9% SL or meso 9% SL were not significantly different from those of untreated controls. However, the relative expression levels of SA pathway-related gene *AsNPR1* showed a 0.41-fold and 0.34-fold decrease in levo 0.9% SL- and meso 9% SL-treated creeping bentgrass, respectively (Fig. 7). At 2 DAT, the relative expression levels of *AsLOX*, *AsERF*, and *AsPOX* in both levo 0.9% SL-and meso 9% SL-treated creeping bentgrass were higher than those in the untreated controls. At 2 DAT, a 6.62-fold increase in the expression of *AsLOX* genes, 1.79-fold increase in the expression of *AsERF* genes, and 2.66-fold increase in the expression of *AsPOX* genes were observed in levo 0.9% SL-treated groups compared to those in the untreated control. Except for *AsNPR1*, the relative expression level of the remaining three genes was higher in the levo 0.9% SL treatment than in the meso 9% SL treatment at 2 DAT.

## Discussion

Over the past few decades, global climate change has exacerbated the frequency and duration of abiotic stresses, such as low temperature and extreme drought. Under chilling and drought stresses, plant productivity is consequently diminished, leading to enormous losses in crop yields worldwide. Chilling and drought stresses have a considerable negative impact on stomatal development and leaf growth, resulting in sustained water and photosynthetic deficiencies [33]. Drought and chilling conditions affect the hydraulic conductance and activity of plant roots, thus creating a water deficit [34]. To solve these problems, the application of PGPR or compounds produced by PGPR is a sustainable and environmentally friendly approach for enhancing the adaptability and tolerance of plants [35]. In this study, we formulated levo 0.9% SL and meso 9% SL with bio-based levo-2,3-BDO and meso-2,3-BDO, which are produced by *B. licheniformis* 4071. We analyzed the morphological, physiological, and molecular effects of bio-based 2,3-BDOs in plants under drought and chilling stresses.

Our results showed that bio-based 2,3-BDOs enhanced the tolerance of plants under drought and chilling conditions. Meso 9% SL treatment induced higher tolerance in tomato plants to chilling and drought stresses compared to levo 0.9% SL treatment. By contrast, levo 0.9% SL-treated turfgrass, creeping bentgrass and Kentucky bluegrass, exhibited higher tolerance to drought stress compared to meso 9% SL-treated turfgrass. Consequently, 2,3-BDO formulations induced abiotic stress tolerance in tomato plants and turfgrass, although the effects of levo-2,3-BDO and meso-2,3-BDO varied between plant types.

Levo-2,3-BDO and meso-2,3-BDO are stereoisomers of 2,3-BDO, but they may possess different action mechanisms in various plants. Therefore, we investigated whether the application of two 2,3-BDO stereoisomers could differentially mediate multiple plant hormone signaling pathways related to plant tolerance of abiotic stress. Most plant hormones have been linked to biotic stress responses [36]. However, endogenous plant hormones can also affect response to abiotic stress. Plants undergo complex processes regulating plant hormone-related and ROS scavenger gene expression in response to abiotic stresses, including chilling and drought stresses. Furthermore, when plants are exposed to environmental stresses, some plant hormones, including SA, ET, and JA, and reactive oxygen species (ROS) participate in the regulation of water uptake [33, 37].

The role of SA in abiotic stress has been widely investigated. SA improves the heat-shock tolerance of mustard and tobacco plants [38, 39]. Exogenous application of SA improves drought tolerance in rice, and acetyl SA decreases the inhibitory effect of drought and salt stress in wheat [40-42]. In addition, hydroponic treatment with SA reduces the effect of chilling injury and may alter the synthesis of ET during chilling in maize [43, 44]. Ethylene is involved in a variety of stresses, including wounding, infection, flooding, drought, and temperature shift [45, 46]. Overexpression of a tomato ethylene response factor (ERF) protein enhanced the tolerance of transgenic tobacco to osmotic stress and low temperature [47]. Ethylene biosynthesis genes (1-*aminocyclopropane*-1-*carboxylic* [*ACC*] *synthase* (*ACS*) and *ACC oxidase* (*ACO*)) were upregulated in resistant and tolerant soybean plants under water deficit conditions [48]. In rice, activation of the JA signaling pathway by methyl JA can enhance drought tolerance [49, 50]. Chilling conditions induce the expression of JA synthesis-related genes, such as *allene oxide synthase* (*AOS*) and *lipoxygenase* (*LOX*), and the synthesis of the bioactive JA form, thereby inducing the expression of cold-regulated genes and enhancing cold tolerance [51]. JA and the JA precursor 12-OPDA promote stomatal closure in *Arabidopsis thaliana* and improve drought resistance [52].

The generation of ROS within the cell may increase in plants injured by drought and chilling stresses [53, 54]. Accumulation of excess ROS causes oxidative damage at the cellular level, disrupting cellular membranes and ionic balance [55, 56]. However, depending on the ROS production and scavenging equilibrium, ROS can also act as protective signaling factors associated with plant tolerance to abiotic stresses. ROS scavenging is an efficient antioxidative defense system, and the activity of antioxidant enzymes may increase in response to oxidative stress [54, 57]. ROS scavenging is a typical protective response to most stresses, including drought, temperature extremes, salinity, UV radiation, heavy metal toxicity, nutrient deficiency, and infection [53]. Although plant hormone-related signaling pathways have been extensively investigated, our understanding of their roles under various environmental stresses is limited and crosstalk between plant hormones and bio-based 2,3-BDOs in plant stress responses still remain obscure.

Treatment with two formulations of bio-based 2,3-BDOs induced expression of several plant hormone signaling pathway-related genes and consequently improved the ability of the plant to cope with chilling and drought stresses. In tomato plants, meso-2,3-BDO treatment induced higher relative expression levels of the SA pathway-related gene *LePAL*, ET pathway-related gene *LeACS4*, and ROS scavenger gene *LePOX* than the levo-2,3-BDO treatment. By contrast, levo-2,3-BDO-treated creeping bentgrass exhibited higher relative expression levels of the JA pathway-related gene *AsLOX*, ET pathway-related gene *AsERF*, and ROS scavenger gene *AsPOX* than the meso 2,3-BDO treatment. These results are consistent with drought tolerance in tomato and creeping bentgrass plants treated with the 2,3-BDOs. The two stereoisomers seemed to induce similar but slightly different mechanisms in the different plant types. After treatment, the expression of both the ET pathway-related gene and ROS scavenger gene increased in tomatoes and creeping bentgrass. However, the expression of the SA pathway-related gene *LePAL* was stimulated by meso-2,3-BDO in tomato plants, and the expression of the JA pathway-related gene *AsLOX* was induced by levo-2,3-BDO in creeping bentgrass.

The crosstalk among various plant hormone signaling pathways in regulating plant stress responses has attracted much attention [58]. This crosstalk participates in the regulation of plant growth and maintaining the balance between defense and fitness [37, 59]. ASM is known to induce systemic acquired resistance and abiotic stress tolerance in plants [60], and exogenous ASM application improves heat or drought tolerance in creeping bentgrass [61, 62]. In tomato plants, ASM treatment induced tolerance under drought stress but had an adverse effect under chilling stress. All tomato plants treated with the SA signaling pathway activator ASM died under chilling stress, suggesting that the application of ASM under chilling stress could have detrimental effects on the regulation of plant growth by disrupting the balance between defense and fitness in tomato plants. Meso 9% SL treatment induced high chilling and drought stress tolerance. In nature, plants often experience various abiotic stresses simultaneously. In a situation where drought and chilling stresses coincide, the bio-based 2,3-BDO formulations show great potential as biostimulants to induce abiotic stress tolerance in plants without side effects. Meanwhile, chilling stress did not cause any damage to creeping bentgrass or Kentucky bluegrass, which is considered due to their being cool-season turfgrasses.

Bio-based 2,3-BDOs could improve the plant tolerance to adverse conditions and act as modulators of plant responses to stress conditions. In this study, the application of levo 0.9% SL in creeping bentgrass and meso 9% SL in tomato plants reduced the deleterious effects of abiotic stresses more than other treatments under drought and chilling stress conditions. Furthermore, except for the increased expression of the ET pathway-related gene and ROS scavenger gene, pretreatment with levo-2,3-BDO in creeping bentgrass and meso-2,3-BDO in tomato plants enhanced JA and SA signaling pathway-related gene transcript expression levels in different ways. Accordingly, bio-based 2,3-BDO isomers could enhance tolerance to drought and/or chilling stresses in various plants through somewhat different molecular activities. Moreover, the bio-based 2,3-BDO formulations exhibited excellent potential as biostimulants to induce abiotic stress tolerance in plants without any side effects, unlike ASM which was used as a positive control in this study. Although bio-based 2,3-BDOs are expected to efficiently induce stress tolerance in plants under complex abiotic stresses, their mechanism in plant responses to chilling and drought injuries is still unclear. Therefore, further studies are needed to assess the effects of bio-based 2,3-BDOs on the tolerance of various plant types in complex abiotic systems with simultaneous and dynamic stresses.

## Figures and Tables

**Table 1:** Primers used for quantitative reverse transcription-PCR.

Gene	Primer sequence (5′–3′)
LePAL	For 5′-ACAGAATTGTTGACGGGTGA-3′ Rev 5′-CCATTCCAGCTCTTCAGACA-3′
LeAOS2	For 5′-CGATTACCTCCGATTCTGGT-3′ Rev 5′-AAATCTTCATCCCACCGAAG-3′
LeACS4	For 5′-AATTGCTCGGAGGTAGGATG-3′ Rev 5′-TTCCTCTTCCATTGTGCTTG-3′
LePOX	For 5′-TGGTATGGCGTAAGTCGGTA-3′ Rev 5′-CTTGGAATCAAAGTCCGGTT-3′
LeUBI	For 5′-GGACGGACGTACTCTAGCTGAT-3′ Rev 5′-AGCTTTCGACCTCAAGGGTA-3′
AsNPR1	For 5′-TCTGCAAGTGCGATGTCCAG-3′ Rev 5′-CCCGAAGACATGGTCTCTCCTA-3′
AsLOX	For 5′-AGGGCTGGTCCTTGATGTCG-3′ Rev 5′-TCTACTACCCCAGCGACAGCAT-3′
AsERF	For 5′-GCAAGGTTGTTGACTGCTGG-3′ Rev 5′-CAACAGTGCCGAAGAAGCTG-3′
AsPOX	For 5′-CTTCGACAACGCCTACTAC-3′ Rev 5′-TTTGCCCATGTTCACCA-3′
AsUBI	For 5′-CAGGACAAGGAGGGCATC-3′ Rev 5′-TTCCTGAGCCTGGTGACC-3′

**Table 2:** Effect of levo 0.9% SL and meso 9% SL on drought stress in tomato plants.

Treatment	Drought tolerance (%)	Fresh weight (g)	Dry weight (g)
Levo 0.9% SL			
Once	25.0 ± 5.1 ^c*^	6.6 ± 0.2 ^b^	1.19 ± 0.02 ^e^
Twice	10.7 ± 5.1 ^b^	6.5 ± 0.5 ^b^	1.21 ± 0.02 ^e^
Meso 9% SL			
Once	50.0 ± 10.1 ^d^	6.5 ± 0.1 ^b^	0.99 ± 0.10 ^d^
Twice	60.7 ± 5.1 ^e^	6.2 ± 0.2 ^ab^	0.82 ± 0.05 ^c^
ASM			
Once	100.0 ± 0.0 ^f^	7.6 ± 0.5 ^c^	0.63 ± 0.15 ^a^
Twice	100.0 ± 0.0 ^f^	9.7 ± 0.2 ^d^	0.72 ± 0.10 ^b^
Untreated control	0.0 ± 0.0 ^a^	6.0 ± 0.1 ^a^	0.61 ± 0.07 ^a^
Irrigated control	100.0 ± 0.0 ^f^	6.7 ± 0.7 ^c^	1.02 ± 0.04 ^d^

Plant groups were treated either once (7 days before stress exposure) or twice (7 days and 1 day before stress exposure). ASM was used as the positive control. Values are presented as means ± SD of two runs with two replicates (four tomato plants/replicate).

*Means with the same letter are not significantly different (Duncan’s multiple range test, *p* < 0.05).

**Table 3:** Effect of levo 0.9% SL and meso 9% SL on drought stress in turfgrass.

Treatment	Creeping bentgrass			Kentucky bluegrass		
	
	Drought tolerance (%)	Fresh weight (g)	Dry weight (g)	Drought tolerance (%)	Fresh weight (g)	Dry weight (g)
Levo 0.9% SL						
Once	86.7 ± 13.3 ^c*^	1.45 ± 0.34 ^c^	0.70 ± 0.23 ^cd^	89.4 ± 6.6 ^f^	1.33 ± 0.39 ^c^	0.39 ± 0.07 ^bc^
Twice	93.3 ± 7.7 ^cd^	1.53 ± 0.24 ^c^	0.40 ± 0.32 ^a-c^	48.4 ± 6.1 ^c^	0.75 ± 0.19 ^b^	0.28 ± 0.08 ^ab^
Meso 9% SL						
Once	46.7 ± 0.6 ^b^	0.90 ± 0.10 ^b^	0.49 ± 0.14 ^b-d^	27.2 ± 9.9 ^b^	0.51 ± 0.07 ^ab^	0.25 ± 0.06 ^a^
Twice	96.7 ± 6.7 ^cd^	1.44 ± 0.24 ^c^	0.67 ± 0.23 ^cd^	42.4 ± 6.3 ^c^	0.70 ± 0.09 ^ab^	0.41 ± 0.03 ^c^
ASM						
Once	56.7 ± 6.7 ^b^	1.32 ± 0.43 ^c^	0.81 ± 0.29 ^d^	63.6 ± 4.1 ^d^	0.53 ± 0.19 ^ab^	0.19 ± 0.02 ^a^
Twice	50.0 ± 12.8 ^b^	1.42 ± 0.22 ^c^	0.48 ± 0.28 ^b-d^	78.8 ± 7.4 ^e^	0.76 ± 0.39 ^b^	0.40 ± 0.23 ^c^
Untreated control	0.0 ± 7.7 ^a^	0.47 ± 0.43 ^a^	0.13 ± 0.02 ^a^	0.0 ± 0.9 ^a^	0.42 ± 0.08 ^a^	0.21 ± 0.06 ^a^
Irrigated control	100.0 ± 0.0 ^d^	1.13 ± 0.19 ^bc^	0.54 ± 0.15 ^b-d^	100.0 ± 0.0 ^g^	1.17 ± 0.32 ^c^	0.58 ± 0.09 ^d^

Plant groups were treated either once (7 days before stress exposure) or twice (7 days and 1 day before stress exposure). ASM was used as the positive control. Values are means ± SD of two runs with two replicates (four turfgrass pots/replicate).

*Means with the same letter are not significantly different (Duncan’s multiple range test, *p* < 0.05).

**Table 4:** Effect of levo 0.9% SL and meso 9% SL formulations on chilling stress in tomato plants.

Treatment	Cold stress	Recovery after cold stress		
	
	Cold tolerance (%)	Cold tolerance (%)	Fresh weight (g)	Dry weight (g)
Levo 0.9% SL				
Once	43.3 ± 6.1 ^de*^	50.0 ± 5.0 ^b^	4.3 ± 0.3 ^c^	0.60 ± 0.14 ^b-d^
Twice	48.6 ± 5.3 ^ef^	76.3 ± 5.8 ^c^	5.5 ± 0.2 ^d^	0.70 ± 0.15 ^c-e^
Meso 9% SL				
Once	38.0 ± 5.3 ^d^	76.3 ± 2.9 ^c^	5.4 ± 0.2 ^d^	0.63 ± 0.06 ^b-d^
Twice	52.1 ± 9.2 ^f^	83.8 ± 11.5 ^d^	6.4 ± 0.3 ^e^	0.73 ± 0.17 ^de^
ASM				
Once	7.9 ± 5.1 ^b^	0.0 ± 0.0 ^a^	2.4 ± 0.1 ^b^	0.43 ± 0.03 ^ab^
Twice	0.0 ± 0.0 ^a^	0.0 ± 0.0 ^a^	1.7 ± 0.1 ^a^	0.34 ± 0.11 ^a^
Untreated control	25.5 ± 2.0 ^c^	56.3 ± 5.8 ^b^	4.2 ± 0.4 ^c^	0.48 ± 0.17 ^a-c^
Control (25	100.0 ± 0.0 ^g^	100.0 ± 0.0 ^e^	6.9 ± 0.3 ^f^	0.89 ± 0.21 ^e^

Plant groups were treated either once (7 days before stress exposure) or twice (7 days and 1 day before stress exposure). ASM was used as the positive control. Values are means ± SD of two runs with two replicates (four tomato plants/replicate).

*Means with the same letter are not significantly different (Duncan’s multiple range test, *p* < 0.05).

**Table 5:** Effect of levo 0.9% SL and meso 9% SL formulations on chilling stress in turfgrass.

Treatment	Creeping bentgrass		Kentucky bluegrass	
	
	Fresh weight (g)	Dry weight (g)	Fresh weight (g)	Dry weight (g)
Levo 0.9% SL	1.97 ± 0.70 ^a^	0.32 ± 0.14 ^ab^	0.44 ± 0.03 ^a^	0.12 ± 0.01 ^a^
Meso 9% SL	1.93 ± 0.39 ^a^	0.36 ± 0.09 ^ab^	0.46 ± 0.05 ^a^	0.13 ± 0.03 ^a^
ASM	1.82 ± 0.31 ^a^	0.30 ± 0.06 ^a^	0.47 ± 0.10 ^a^	0.13 ± 0.03 ^a^
Untreated control	2.06 ± 0.43 ^a^	0.39 ± 0.06 ^ab^	0.43 ± 0.08 ^a^	0.11 ± 0.02 ^a^
Uninoculated control	2.28 ± 0.57 ^a^	0.51 ± 0.19 ^b^	0.42 ± 0.08 ^a^	0.11 ± 0.02 ^a^

ASM was used as the positive control. Values are means ± SD of two runs with two replicates (four turfgrass pots/replicate).

*Means with the same letter are not significantly different (Duncan’s multiple range test, *p* < 0.05).
